# Strengthening the Healthy Adult Self in Art Therapy: Using Schema Therapy as a Positive Psychological Intervention for People Diagnosed With Personality Disorders

**DOI:** 10.3389/fpsyg.2019.00644

**Published:** 2019-03-22

**Authors:** Suzanne Haeyen

**Affiliations:** ^1^GGNet Mental Health Centre, Apeldoorn, Netherlands; ^2^Department Arts Therapies, HAN University of Applied Sciences, Nijmegen, Netherlands; ^3^KenVaK, Research Centre Arts Therapies, Heerlen, Netherlands

**Keywords:** art therapy, positive mental health, positive psychological interventions, schema focused therapy, healthy adult mode, personality disorders, borderline personality disorder

## Abstract

This article reviews recent theoretical contributions and studies about art therapy that attempt to capture aspects of healthy ego functioning and presents an inventory of art therapy interventions to strengthen the Healthy Adult. It discusses how art therapy can offer interventions that focus specifically and directly on the integration and growth of the whole person, and which interventions are suitable for this purpose in the treatment of people diagnosed with severe personality disorders. A link is made between the Healthy Adult as a familiar concept from Schema Therapy, and Positive Psychology, with its focus on well-being, strength and positive affect. On the basis of our present knowledge, available studies and experiences, art therapy seems promising in contributing to the development of Healthy Adult functioning. Art therapy addresses the different areas of healthy adult functioning. Specific art therapy interventions are discussed on a concrete level. Art therapy appears to offer ways to strengthen the Healthy Adult and helps people to free themselves from destructive patterns and to work on satisfying their basic needs as independent and responsibly functioning individuals in a positive connectedness with themselves and their surroundings. The strength of art therapy may be the experiential level and the appeal to the capacity to play, to flexibility, and to be creative. This makes experiences easier to reach, and developing from there into a Healthy Adult mode is possible in a manner that is more felt than thought. Creativity can be regarded as the ability of the Healthy Adult to be flexible and to find different solutions to a problem. This appeal of art therapy fits well the therapeutic goal of empowerment and well-being, which is at the heart of positive psychology. More research is needed to verify the effects and working mechanisms of art therapy interventions.

## Introduction

Art therapy appears to offer ways to strengthen healthy adult functioning because it provides an opportunity for people to come in contact with their own feelings and needs as well as those of others. In art therapy interventions processes are often more felt than thought, and approaches appeal to the integration and growth of the whole person. Art therapy might help people to free themselves from destructive patterns and to work on satisfying their basic needs as independent and responsibly functioning individuals in a positive connectedness with themselves and their surroundings. The aim of this article is to review recent theoretical contributions and studies about art therapy that attempt to capture aspects of healthy ego functioning and it presents an inventory of art therapy interventions to strengthen the Healthy Adult as a familiar concept from Schema Therapy. It discusses how art therapy can offer interventions that focus specifically and directly on the integration and growth of the whole person, and which interventions are suitable for this purpose in the treatment of people diagnosed with severe personality disorders. A link is made between Schema Therapy and Positive Psychology, with its focus on well-being, strength and reinforcing positive affect.

The following databases for literature on art therapy were consulted (date 22.02.2019): PsychINFO, Academic Search Complete, MEDLINE, CINAHL, NARCIS, ERIC and Psychology and Behavioral Sciences Collection (EBSCO), Social Sciences Citation Index, ScienceDirect en Science Citation Index. Keywords were: “personality disorder^∗^” or “personality patholog,^∗^” “art therap^∗^” or “arts therap^∗^” or “creative therap^∗^” in all field, and “schema therapy” or “positive.” An additional search was based on the key words: “positive art(s) interventions.” The search was delimited by the term: age 18 up. This peer reviewed research literature was supplemented by descriptive literature; handbooks and descriptions of expert opinions.

First, a link is made between the Healthy Adult mode, Schema-focused therapy, Positive Psychology, personality disorders and the search for specific interventions. Second, based on the hypothesis that art therapy interventions can contribute to the development of Healthy Adult functioning in patients diagnosed with personality disorders cluster B/C, the focus is on art therapy and personality disorders and on healthy ego functions linked to art therapy interventions in general terms. Third, specific art therapy interventions to strengthen the Healthy Adult are discussed on a concrete level. Throughout the text clinical case examples illustrate the therapeutic use of arts interventions for the purpose of strengthening the Healthy Adult in the treatment of people diagnosed with personality disorders. Informed consent was obtained from participants to include their artwork.

## Schema-Focused Therapy, the Healthy Adult Mode and Positive Psychology

Schema-focused therapy, developed for people with personality disorders, starts from the premise that people’s actions, feelings and thoughts are guided and controlled by schemas (Young, 1994; Farrell et al., 2014). Schemas contain collections of knowledge gained in the course of one’s life, a large part of which is implicit. This implicit knowledge cannot be put into words directly, and can only be reconstructed indirectly. Schemas can be active in a number of ways. People can surrender to their schema, their response to a schema can be to avoid painful feelings that are part of their basic schemas or they can overcompensate painful basic schemas by doing the opposite of what the schema suggests (schema overcompensation). For example, they might develop narcissistic self-esteem as overcompensation for their painful sense of inferiority. Schemas are not active all the time. A person may be able to function normally in a great many situations, and then suddenly panic and become clingy when faced with the possibility of being abandoned. Then a schema is activated that takes over and controls the patient’s thoughts, feelings and actions. A schema that is so controlling is called a schema mode. Young describes eighteen basic schemas and four main types of schema modes (Young, 1994; Young et al., 2003). These types of schema modes are: (1) the healthy adult mode, (2) the child mode, in borderline patients often the “angry child” or the “vulnerable child,” (3) the maladaptive protective mode, for example the “detached protector,” disconnected from feelings, and (4) the internalized parent mode, in borderline patients often the punitive parent.

Psychotherapy formulated in schema mode terms focuses on correcting the schemas from childhood and allowing them to develop, switching off the protective states (because they are dysfunctional), and replacing the internalized parent schemas (also dysfunctional because they are demanding, punitive or rejecting) by healthier schemas (e.g., Arntz, 2011, 2012; Farrell et al., 2014). The ultimate object of Schema Therapy is to broaden and strengthen the Healthy Adult. According to current thinking, when you switch off the destructive modes and build up a solid therapeutic relationship, the Healthy Adult in the patient will come more and more to the fore. “The goal is for the patient to internalize a Healthy Adult mode, modeled after the therapist, that can fight schemas and inspire healthy behavior” (Arntz and Bögels, 2000; Young et al., 2003). The Healthy Adult does not actually need to be approached as a mode, but can also be viewed as the integrated person the patient is. The definition as used in this article: “As Healthy Adults, patients use their psychological capabilities to free themselves from ingrained destructive patterns so that they are sufficiently able to work on satisfying their basic needs as independent and responsibly functioning persons in a positive connectedness with themselves and their surroundings” (Claassen and Pol, 2015).

Very little is said in the literature about specific interventions to broaden or strengthen the Healthy Adult. Art therapy is quite often used in the clinical or outpatient treatment of personality disorders. Art therapy in patients with personality disorders of clusters B or C came forward as effective. Art therapy showed several beneficial effects for personality disorder patients in a series of studies, including a randomized controlled trial (Haeyen, 2018a). First, it is effective in reducing mental illness: in reducing general mental disfunctioning, and in reducing specific symptoms of personality disorders of clusters B or C like early maladaptive behaviors or states (impulsivity, detachment, vulnerability and punitive behaviors). Second, it enhances adaptive modes (pleasant feeling and self-regulation) and ameliorates positive mental health; it increases well being and other positive measures. Finally, unpleasant inner thoughts, feelings, and physical sensations appear to be more easily accepted. The strengths of art therapy could be: the direct, experiential therapeutic entry, the possibility for the art therapist to approach the patient and his problems in an indirect way and the qualities involved in working with art materials, the art process and art product.

Healthy Adult functioning is connected to positive emotions, that can be defined as desirable adaptive experiences of typically brief multiple-component response tendencies that subjectively feel good, and both signal and produce optimal functioning (Fredrickson, 1998, 2004, 2013). There is a rapidly growing interest in psychological well-being as outcome of interventions. Numerous interventions have been developed with the aim to increase psychological well-being. The effects on psychological well-being measured as coherent outcome (based on theory-based indicators developed by Ryff) examined across studies was moderate to small (Weiss et al., 2016). A central aim of these interventions is to enhance positive psychological functioning. None of these studies concerned an art therapy intervention. Given the outcomes in recent studies of increased positive mental health with large effect sizes (Haeyen, 2018a), art therapy could be a very promising intervention to strengthen Healthy Adult functioning.

In addition to the usual nature of treatment, which is complaint-oriented, Positive Psychology also focuses on well-being, strength and reinforcement of positive affect (Seligman and Csikszentmihalyi, 2000; Seligman et al., 2006; Bohlmeijer et al., 2013a,b). This approach is well adapted to the present broad trend toward thinking in terms of a patient’s strength rather than taking a one-sided approach aimed at reducing the patient’s complaints. Art therapy is a unique kind of psychotherapy that uses the process of making and viewing art within the context of a helping relationship to increase well-being for patients of all ages (Wadeson, 1980; Rubin, 1999; Malchiodi, 2011).

## Art Therapy and Personality Disorders

Art therapy is a treatment form for people with psychiatric disorders and psychosocial problems in which methodical use is made of an experience-oriented working method. The systematic use of art assignments, art materials and techniques in these areas forms the vehicle for professional practice (e.g., Rubin, 1999, 2001; Schweizer et al., 2009; Malchiodi, 2011; Gussak and Rosal, 2016). In the process, the patient’s problems can come forward, leading to experiences that have an effect on the disorder. Art therapy is also about positive experiences, about play, joy and creating. It concerns the value of creating emotional expressive artworks that increase intersubjective knowledge through empathic resonance, a key to psychological integration, growth, and change (Chilton et al., 2015). Art therapy from a positive psychology perspective focuses on expanding what is working in the patient’s life. The theory of positive emotions in positive psychology literature always also recognized the importance of negative emotions recognizing that both kinds of emotions have evolutionary survival value (Fredrickson, 1998, 2004; Fredrickson and Losada, 2005). Even more, working with patients with serious and long existing problems such as personality disorders, cannot solely focus on positive aspects. It does require making contact with what is experienced (often difficult emotions) or with the avoided experiences. The object is to achieve both emotional and cognitive change. Change may involve development, or stabilization and acceptance. Change is sometimes seen in a social and physical sense as well (e.g., Malchiodi, 2011; Gussak and Rosal, 2016).

Art therapists diagnose in their own professional field. They contribute to treatment by recognizing, influencing and changing people’s behavior (FVB, and GGZ Nederland, 2012; Haeyen, 2018a). Art therapy works with visual materials and techniques, with emphasis on how the materials are experienced and used, or how the art media are given shape. Art therapy is fundamentally about experiencing, expressing and emotionally structuring patterns of feelings, thoughts and reactions through the art medium. This allows patients to gain insight and learn from corrective experiences through discovery, improvisation, experimentation, modeling and shaping, or intuitive actions in the moment (e.g., Lusebrink, 1989; Rubin, 2001; Hinz, 2009). Learning by doing, patients can create meaningful images and experiences. Patients often experience art therapy as a direct route to deeper emotional layers – a personal confrontation with themselves and their own patterns of thinking, feeling and acting in a relatively safe situation. Patients often perceive art therapy as safe, dished out in small doses – a way for them to reach feelings pertaining to their therapeutic development (Haeyen, 2018a,b). The materials, techniques, colors and images offer a wide range of specific possibilities within art therapy. Art therapy focuses on creativity; this has much less to do with creating esthetically pleasing results than with toying with possibilities, being flexible, devising more than one solution, trying things out (e.g., Lusebrink, 1989; Rubin, 1999, 2001; Hinz, 2009; Schweizer et al., 2009; Malchiodi, 2011; Gussak and Rosal, 2016). Patients whose development has slowed or stopped often have difficulty with this. Their mental resilience is absent, low, or greatly reduced. They are caught in their patterns of emotions, thoughts and actions and feel no room to investigate, no scope to examine. Creative abilities have gone into hibernation or are blocked. In art therapy, we try to nurture this creativity, stimulate it, and develop it. The works produced by patients make these processes visible and concrete (Haeyen et al., 2018).

In the treatment of personality disorders, it is clear that art therapy makes use of Schema Therapy and that, more and more often, in both clinical and outpatient treatment, therapists work along these lines. Experts in the field as well as the Dutch national multidisciplinary guideline for personality disorders [*National Advisory Committee Regarding Multidisciplinary Guidelines for Development in Mental Healthcare*] assume that art therapy can be integrated in Schema Therapy, and that it is a useful complement to it (*Landelijke Stuurgroep Multidisciplinaire Richtlijn ontwikkeling in de GGZ [National Advisory Committee Regarding Multidisciplinary Guidelines for Development in Mental Healthcare]*, 2008; Schweizer et al., 2009; Haeyen, 2011, 2018a,b; Van Vreeswijk et al., 2012a,b).

Various approaches can be taken in combining Schema Therapy with art therapy. Art therapy can start from the schema domains or the modes. It is possible to work directly, indirectly or semi-directly with the schema terminology. Patients can represent their own perceptions and gain new experiences while making art works. Art therapy can trigger a variety of different modes (Van den Broek et al., 2011; Van Vreeswijk et al., 2012a). Patients can give shape to and reflect on modes, whether in dialogue with themselves or in contact with others. Although a person’s past is considered, the effect in the here and now continues to be the central focus. In relation to the use of language and creation of meaning, schema theory offers clear representations in the form of the modes, and the schema domains become very recognizable in the process of making expressive art works.

Art therapy is well-adapted to Schema Therapy because Schema Therapy makes use of experiential treatment techniques (e.g., Arntz, 2011, 2012; Farrell et al., 2014; Reubsaet, 2018). Experiential techniques focus on the client’s current feelings, perceptions, and bodily sensations. The therapeutic intervention is designed to promote the client’s ability to make his or her own choices. Experiential techniques enhance awareness and promote experience and expression of emotionally laden material (Haeyen, 2018a,b). Becoming aware of and integrating disowned aspects of experience or aspects that are in conflict is an important theme. The goal is to discover and explore what one is experiencing and use this to inform choice and action (Greenberg et al., 1989). Experiential techniques (such as imagery, role playing and the multiple chair technique) are also used in Gestalt Art therapy (Rhyne, 1970; Amendt-Lyon, 2001; Schweizer et al., 2009) and art therapy in a broader perspective. These techniques are used so that patients will experience and express feelings and thoughts, clarify them, make links between recent events and experiences or perceptions from their childhood, and identify schemas. A new schema can take shape on the basis of the client’s experiences in art therapy (Haeyen, 2018a). Because the information is not actually provided consciously and verbally but in a non-verbal experience, the new representations that arise are more felt than thought (Arntz and Bögels, 2000). A recent multicenter study (Bamelis et al., 2014) showed that Schema Therapy using experiential exercises was more effective than Schema Therapy in which the focus was on talking, followed by an explanation without the use of exercises. Little is known about how art therapy can be enlisted as a Positive Psychology Intervention (PPI). Only a few studies are available, and a small number of articles that touch on this.

Based on the hypothesis that art therapy interventions can contribute to the development of Healthy Adult functioning in patients diagnosed with personality disorders cluster B/C, the following section focuses on the link between healthy ego functions and art therapy interventions.

## Healthy Ego Functions Linked to Art Therapy Interventions

Arntz and Jacob (2012) include healthy ego functions in their description of the Healthy Adult. These healthy ego functions involve being able to make healthy choices and striving for well-being for yourself and for others. Healthy ego functions are (1) Personality integration and formation of self-image; (2) Healthy emotion regulation; (3) Coming in contact with one’s own feelings and needs as well as those of others; (4) The healthy internal dialogue; (5) Reality testing and assessment of situations, conflicts, relationships; and (6) Seeking enjoyment and need fulfillment in a mature manner. How can art therapy help strengthen a number of healthy ego functions? The following text will elucidate how art therapy can help strengthen these six basic healthy ego functions.

### Personality Integration and Formation of the Self-Image

**FIGURE 1:**
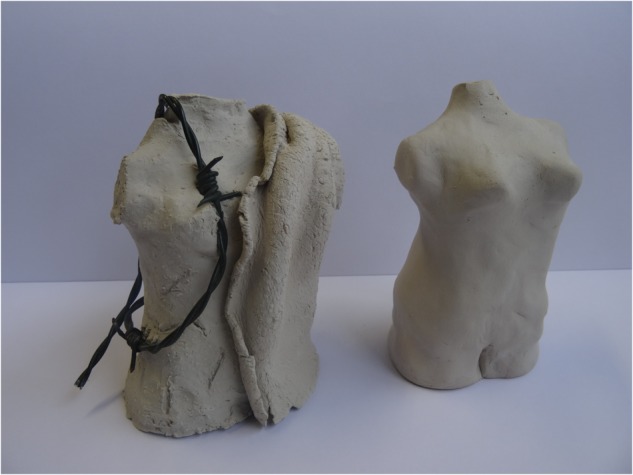
Torsos.

In art therapy, the inner self becomes visible, tangible and concrete through the work produced (e.g., Rubin, 1999, 2001; Schweizer et al., 2009; Malchiodi, 2011; Gussak and Rosal, 2016). It may be a conscious or unconscious process, and the meaning is always determined in a dialogue with the client. Patients learn to reach their emotions, and exhibit increased cohesion and integration of their personality. Both integration and fragmentation take place at a deep level of personality. For example, personality integration takes place when opposing feelings, thoughts and behaviors can come together in a single coherent representation (Rubin, 2001; Haeyen, 2018a). The art work confirms what is present, or makes unseen qualities visible. A person’s development in the therapeutic process becomes manifest in art work, and he or she can draw on this experience. Self-reflection takes place on the basis of the expressive art work and the art therapy assignments (e.g., Malchiodi, 2011; Gussak and Rosal, 2016). Here art therapy is focused on improving a person’s self-image; this takes place at a somewhat higher level of personality. Although partly related to coherence, it is primarily about content – a positive or negative self-image. The art product allows patients to take some distance, so that they can contemplate themselves and their own dynamic on the basis of a concrete art work or a series of assignments. They can choose to let go of the art work, or put it away somewhere, as the bearer of what it is that bothers them and occupies their mind (Haeyen, 2018a,b). A person can bring a situation to a full or partial conclusion by leaving the work somewhere. He or she can practice dealing with things differently, and experience how it feels to do things in different ways. All these aspects stimulate patients to perceive themselves as *one whole* and as *always the same person*, even if vehement emotions are elicited; this is a characteristic of mentally healthy individuals. People with a borderline personality disorder, for example, can become completely preoccupied with a violent emotion and lose themselves in it (Van Vreeswijk et al., 2012b). In that case, the person’s sense of continuity can become impaired (Young et al., 2003). The following art product (see Figure 1) and the accompanying quote show how a negative and impaired awareness of body is studied and how a more positive experience is sought in working expressively via interventions focused on the tactile experience and the shaping of a more positive self-image.

“I made these torsos to give expression to my body awareness in art therapy. On the first torso, I used barbed wire to represent my damaged feelings. I was encouraged to challenge myself and to do the assignment again, but now with love and self-compassion for my body.” (woman, age 30)

The intervention used by the art therapist in this case involved allowing the client to work “blind” in the second variant. Here, this means that the client worked the clay and gave shape to her torso with her hands in a dark plastic bag, so that she could not see what her hands were making. This brought her closer to the tactile experience and offered her less control. The client deliberately worked the clay gently, lovingly, mildly with soft pressure, slow hand movements and working from the fingertips. While working, this client was touched by the fact that she really fully experienced this different manner of working and realized how unusual this was for her. When she took her work out of the plastic bag and could see it, her critical side, her punitive/critical parent mode presented itself. She expressed herself on the shape in critical terms: “Ugly, wide, exactly how I look at my body.” In the follow-up discussion, the group reflects on the process of making, the experience and the difference with what happened when they could see their work. It can be discussed which different modes were active, and what was new and positive about the experience, for example in this case, that she allowed herself to feel something, she could give shape to her own torso gently, and she allowed her emotions to be present. The reflective discussion afterward is important for validation for being brave enough to take part in an experiment.

### Healthy Emotion Regulation

It is assumed that the Healthy Adult can be strengthened through awareness. In art therapy, states of mind can be triggered by what happens during the art process and the interaction with the visual materials. In a pilot study (*n* = 10) as part of a 3-year randomized controlled trial (RCT) (*N* = 120) at seven in-patient hospital clinics, significant results were found for the effectiveness of arts therapy, including art therapy, in eliciting emotional states in patients with a cluster B personality disorder (Van den Broek et al., 2011). Past experiences sometimes emerge spontaneously in response to the attraction inherent in materials and assignments. Reflecting on this leads to awareness: What exactly moves me? Where does this come from? How can I deal with it? Do I want to deal with it? A mentally healthy person can experience more than one state of mind simultaneously, can be aware of both anger and grief and reflect on these states. Together, several states of mind are adequate in the situation at hand; they form a flexible and conscious reaction (Young et al., 2003). Patients are not overwhelmed by negative emotions in relation to small problems (Van Genderen et al., 2012). The following quote shows how emotions that are linked to past experiences can be triggered and how a person can reflect on this.

“Feeling the clay is something I find gruesome. Then I start to talk, to babble, just to avoid the emotion... What I really want is to get away from it as quickly as possible. I dared myself to spend some more time reflecting on it. Then I made a pile of bits and pieces. That fits how I have been feeling recently, things are becoming too much for me. Everything just piles up... I find clay awkward, difficult, because I get dirty when I work with it and it feels creepy. It brings me back to earlier days. My mother would never let me get myself dirty and if I did, then I was in trouble... then I got spanked. Now that I’ve been thinking about it and reflecting on it, I think that the fear I had back then has been triggered.” (man, age 42)

This patient described how he was able to increase emotional regulation, he challenged himself to experience the negative feelings associated with clay and the early childhood experiences and memories that come along with this. A more distanced analysis of one’s negative feelings in the reflective discussion afterward can reduce negative affect or can make it possible to further create an image or a reconstruction of this experience which requires a degree of psychological distance (Dalebroux et al., 2008).

### Contact With Own Emotions and Needs as Well as Those of Others

**FIGURE 2:**
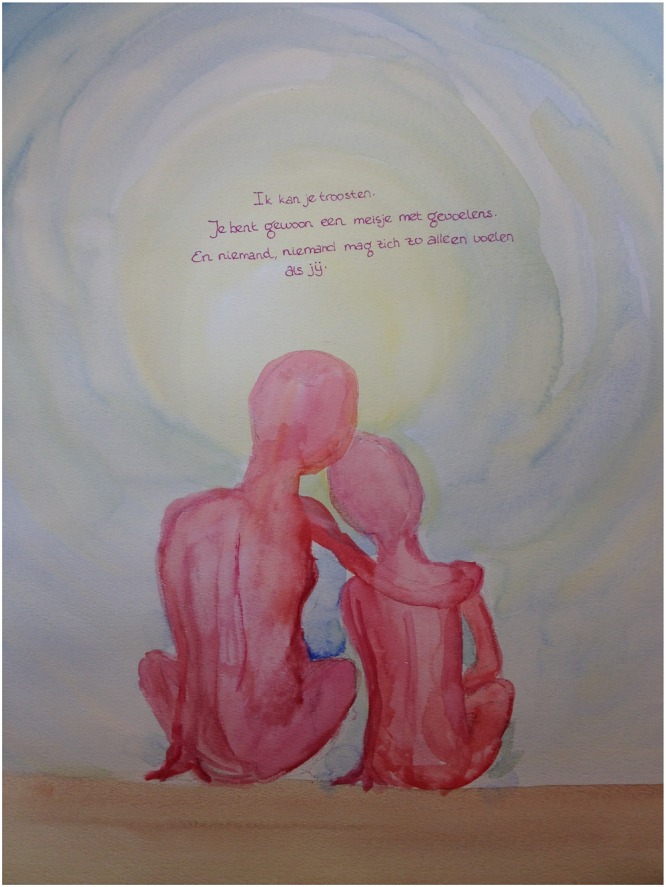
Consolation.

Art therapy is strongly focused on connecting to and attuning to the needs and capabilities of patients: What do they find agreeable? What could they do or make? What sorts of things might they experiment with; are there alternatives they could toy with, perhaps consider? What are their possibilities, what is impossible for them, and how can they find a way to give shape to their life that is meaningful for them? (e.g., Rubin, 1999, 2001; Schweizer et al., 2009; Malchiodi, 2011; Gussak and Rosal, 2016). A positive challenge forms the basis for further development. Patients draw on the primary process of sensory experience via art therapeutic interventions (Hinz, 2009; Darewych and Riedel Bowers, 2018). Language is not yet part of it, nor is it immediately necessary. Language is an abstraction. Themes can be explored via this primary process without immediately addressing them through language. The specific art therapy medium has directness in calling forth the exploration of this primary process. For instance, a need for consolation (see Figure 2) can be shaped by working with soft materials. During the process, an emotional working through can take place in that the client’s need is fulfilled by the work in art therapy (e.g., Lusebrink, 1989; Rubin, 2001; Hinz, 2009). Other names for it are a reconstructive working method or a corrective, emotional, or subjective experience (e.g., Gunther et al., 2009; Haeyen, 2018a,b; Haeyen et al., 2018). Some patients find this experiential method very tense, gripping, or difficult, such as patients with personality disorders who tend to avoid experiences and emotions because they are afraid of losing control. Despite the fact that it may be very relevant to their therapeutic process, they perceive a high threshold when they are invited to “enter into” experiences. The art exercises offered to them individually or as a group allow patients to become aware of their own feelings and needs and those of others. Gaining insight into their own coping mechanisms creates room to choose a different response. In art work during art therapy, there is plenty of scope for contact with child modes. They can be evoked by emotional reactions to materials and assignments because they invite play (Gunther et al., 2009; Van den Broek et al., 2011). Protective modes can be recognized, for example, when a client produces works that are unfeeling or insensitive, or avoids emotions in some other manner, consciously or unconsciously. Parent modes come forward, for example, if patients take a severe and punitive view of their own work: the product is judged and found wanting; it is not good enough (Haeyen, 2015). Patients can practice opposing the demanding and punitive parent (Arntz and Bögels, 2000; Young et al., 2003; Farrell et al., 2014). Emotions and manners of expression that are related to child modes can be validated, acknowledged and made important. The Healthy Adult is in contact with his or her own emotions and needs and those of others. Healthy Adults can recognize their own emotions and needs, as well as those of others, and they know how to strike a good balance between them (Van Genderen et al., 2012). The following quote shows how an emotional need can be recognized and validated by making an image which helps to confirm this need. The expression of this need and the image of the embrace of herself worked as a way of self consolation.

“As soon as the three-day (part-time) treatment started, a sociotherapist asked me, when I was upset, what it was I needed. “How should I know?!” I cried out. And I really had no idea, I just felt lost and adrift.’ “Consolation?,” he suggested … Consolation. It’s that simple. But it had never occurred to me, because when I was little, no one ever consoled me, and I never asked for it, because I was convinced that I was just acting up, being silly.

It took me until I was 39 and had been in art therapy for three years before I managed to hug the child in me, the child who felt so lost and alone. And doing this was very scary. Confrontational.

It was only after I made this work, the child being embraced with a brief text from a letter that I began to take her seriously, and at the same time my past as well. It cannot be denied that terrible things happened.

(…) It is still hard for me to feel that I have actually been consoled. But the therapist encourages me, and it feels good to produce something different for a change, something milder than the basically destructive work that I usually make.” (woman, age 39)

As this image and quote shows, making a consoling painting like this helps to develop and practice self-compassion.

### The Healthy Internal Dialogue

In art therapy, different sides of a person can be represented and linked to one another. This encourages the internal dialogue, as is supported by many studies and descriptive literature (e.g., Neumann, 2001; Malchiodi, 2011; Gatta et al., 2014; Czamanski-Cohen and Weihs, 2016; Haeyen, 2018a). The dialogue between the various sides of oneself can take place both literally and figuratively – for example, by asking what the various sides of the person say to each other: “If these clay figures (one of which, for example, represents the angry child mode and the other the punitive parent) could say something to each other, which of them would, and what would he or she say?” This type of follow-up discussion, in which the client stays very close to the expressive experience, can often be surprisingly revealing. Being able to conduct an internal Socratic dialogue is regarded as specific evidence of the presence and growth of the Healthy Adult (Arntz and Jacob, 2012). In recent research of how patients view the effect of art therapy, the internal dialogue that arises through the art work is explicitly mentioned (Haeyen et al., 2015; Haeyen, 2018a). Patients say that in art therapy, they experience a different dynamic than in other forms of therapy, which almost always work with direct interaction. Interactions then follow one another in fairly quick succession, and people sometimes become absorbed in thoughts about the communication process itself. Patients perceive the process in art therapy as more gradual, calmer. In art therapy, and certainly in individual assignments, people concentrate more on themselves, allowing for more uninterrupted internal dialogue. This gives them a better overview and greater control. Thanks to this different dynamic, it is sometimes possible in art therapy to have more and/or better contact with deeper feelings (Haeyen et al., 2015). Sometimes, for this very reason, patients are overcome by their fear of emotions and fear of loss of control. Thanks to the improved contact with deeper feelings, by pursuing a healthy internal dialogue, a more stable sense of self can emerge as well as better contact with oneself and others. In order to gain control of the other modes, it is important that the Healthy Adult confirms the vulnerable child mode, consoles and sets limits to the angry child mode, and neutralizes the punitive parent mode (Muste et al., 2009).

### Reality Testing and Assessing Situations, Conflicts, Relationships

Working with experiential art therapy interventions consistently calls for a process of becoming involved and participating on the one hand, and taking distance and reflecting on the other hand (Schweizer et al., 2009). This encourages patients to make contact with themselves, with what is going on, with what they are concerned about, and with others. Immediately after this, patients need to take distance, look at what is happening and determine their point of view. It is precisely this process of involvement and distance that allows a client to develop (Haeyen, 2018a). Distancing themselves faces patients with reality testing in concrete terms. A client who says at the start of therapy, “I can’t do this, I’m not creative!” is confronted with the fact that he or she has created something that very likely exhibits individuality. This seems to have the strongest effect in a group situation (e.g., Schweizer et al., 2009; Malchiodi, 2011; Gussak and Rosal, 2016). The group members can see each others’ works in all their diversity, showing the characteristic traits of the person who made them. What people make always shows a certain style or handwriting of their own. Reality testing and assessment of situations, conflicts, relationships occur concretely in a range of expressive forms of cooperation. Patients work on an assignment in groups: this challenges each participant to interact with the others, who may well have different interests and preferences (e.g., Schweizer et al., 2009). The assignments can be organized so that specific aspects will come up. For example, an assignment can focus on congenial cooperation, on dealing with autonomy versus working jointly and dealing with conflicts. Experimenting with this and working constructively and solution-oriented is encouraged. On a very practical level, it is about organizing and paying attention to the work environment within the working relationship with the therapist and the other group members.

**FIGURE 3:**
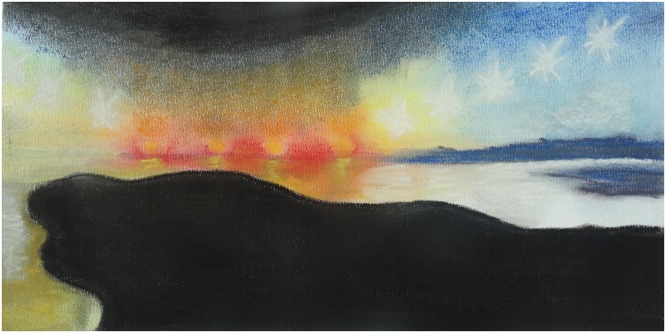
Emotional landscape.

Thanks to the art therapy situation, old, maladjusted schemas can come to the surface (Gunther et al., 2009; Van den Broek et al., 2011; Haeyen, 2018a). These schemas emerged because a child’s basic needs were threatened. Working on becoming aware of these old destructive patterns is often made difficult by blockades: alienation or isolation of affect. Due to cognitive distortion, certain coping reactions (surrender, avoidance and overcompensation) and inadequate lifestyles, people have less contact with their own emotions (Young et al., 2003). In art therapy, affect is sought out and distortions or non-constructive coping reactions are challenged and if possible, corrected. Figure 3 and the accompanying quote show how art therapy can lead to awareness of emotions, and how patients can look at this from a greater distance.

“I went into therapy because I was deeply depressed. Everything was emotionless and I just wanted to die. I was never angry or sad, I didn’t allow myself to be. Fear just did not exist. And if those emotions were there at all, I did not recognize them as such. I didn’t always feel comfortable among my fellow group members, who seemed to be on edge all the time, emotionally speaking.

Until one day when I was exhausted and during art therapy, or actually in the follow-up discussion of our work, I got angry with someone in my group. Before that day I would ask myself: what am I doing here, I make something, but do I actually *do* anything? But then suddenly the penny dropped, and I realized that on that day, in my art work, I had vented my frustration with his attitude. And so anger was the first emotion I recognized. From then on, it would flare up several times a day over seemingly nothing. And then I did identify with my fellow group members…

Because all my life I had not acknowledged my emotions, after that there were a great many intense flare-ups. Even being happy was dangerous, and it still is, because it is unknown territory, things I don’t know how to deal with. This assignment shows the perpetual mood swings. My mood still changes several times a day, but since I recognize emotions and know that anger and fear are allowed and that they do subside (although slowly), everything is more fluid, instead of the uncontrollable and intense ups and downs.” (woman, age 52)

### Seeking Enjoyment and Need Fulfillment in a Mature Manner

In art therapy, complaint-oriented and strength-oriented largely seem to go hand in hand: the combination of affinity with the activity – painting, drawing or spatial design (strength) – while also working on complaints. Working complaint-oriented means practicing what is difficult, resolving blockages by activating ourselves literally and figuratively. Working with objectives and giving expression to an often overwrought inner world can then focus on both strengths and complaints at the same time (Haeyen, 2018a). Working expressively in therapy can also mean taking pleasure in it, relaxing. When patients start working expressively on the basis of their affinity, they may quickly move out of their comfort zone into a development zone. It is characteristic of art therapy that it works with play-related methods, assignments and techniques that may be very similar to experiences in day-to-day reality, but from which patients can distance themselves in pretend situations, so that the experience becomes accessible and can be evoked again later. Imagination, fantasy and play can create room for patients to explore these situations in therapy (Kosslyn, 2005; Farrell et al., 2014; Faranda, 2016). By varying the degrees of distance and closeness, patients can be activated and set to work on their objectives in a safe environment. The art therapist seeks interventions that are attractive to patients, that utilize their strengths, increase their capacities and are in line with the treatment plan. This makes it possible for patients to experience the fun factor while working on problems indirectly, through play or pretend situations. It lightens the idea of being in therapy and is linked to a positive affect, to some degree balancing the weight of the problem or complaint.

The foregoing descriptions of art therapy as being helpful and effective in seeking enjoyment and need fulfillment in a mature manner correspond to the findings in a recently conducted randomized controlled trial (RCT; *N* = 74) based on an art therapy intervention (Haeyen et al., 2018a,b). The object of this RCT was to evaluate the effects of the art therapy intervention developed for people diagnosed with personality disorder. The patients in the experimental group showed improved mental health at symptom level as well as adaptive schema modes which concern being able to experience pleasant feelings, to feel spontaneity and to able to self-regulate. The effect sizes were large to very large.

## Specific Art Therapy Interventions

In the preceding paragraphs, the various healthy ego functions were linked to how art therapy can help strengthen them. The next paragraphs discuss the specific art therapy interventions to strengthen the Healthy Adult on a concrete level.

### Interventions Focused on Self-Acceptance and on Training Compassion

“This is what you are making, it’s yours. First of all, can you leave it as it is / leave it whole?” Creating something yourself is often accompanied by uncertainty, which is normal. However, for people with personality disorders, this uncertainty can take on huge proportions (Haeyen, 2015). Many art therapists working with this target group will recognize that patients sometimes can hardly bear to look at what they have made, and just want to throw it out, leave it behind somewhere or talk about it in deprecating terms during the follow-up discussion, which often takes place in the group. In art therapy patients are encouraged to take care of their own work, to put it away carefully or even to cherish it. Interventions are always focused on looking *non-judgmentally*, on dealing with their own persistent patterns of self-criticism, and on awareness of their own self-deprecating thoughts by pointing them out during the process and in the discussion. The art product can stimulate emotional perception, self-insight and a more observant or sometimes also more down-to-earth perspective. The art product can offer a bridge for communication, especially when fear or resistance is high (e.g., Rubin, 2001; Lefevre, 2004; Moschini, 2005).

During the process and the follow-up discussion, problems with self-acceptance and self-compassion mostly become manifest. While they are handling the materials and in the follow-up discussion, patients can practice dealing with these matters differently, which can lead to insight. Successful experiences in making art work can encourage patients to change their feelings and thoughts; for example, “I’m not good at anything” can be changed by experiences of success in art works into “I can do this!” (Schnetz, 2005; Wilkinson and Chilton, 2013). In concrete terms, this may mean that patients choose a work with which they are the most satisfied or the least dissatisfied, and put it in a frame. Or it can mean giving shape to the negative voice they hear inside themselves. Then this “*tormentor*” can take on physical form. Looking at it from a distance and sharing it with others often leads to a somewhat different view of it. Patients can realize that the way they look at something is defining, and why, or they are able to look at their own work from a somewhat more detached perspective (Haeyen, 2018a). Previously, they were one, fused, with the negative voice, but now they can take some more distance from those thoughts. This process is called defusing from emotions (Hayes, 2000), as opposed to merging or fusing with them.

### Interventions Focused on Giving Expression to the Experiential World

This can be about the situation here and now, or the experiential world over a longer period of time – for example, an expressive tale of a person’s life story. A focus on the *here and now*, for example, is aimed at reinforcing and/or increasing positive experiences. Darewych and Riedel Bowers (2018) presented some positive arts interventions based on thid principle. One way of reinforcing and/or increasing positive experiences is by creating a positive diary. Positive feedback, perhaps in the form of illustrations chosen for the person by others, can be part of this. People are often very pleased with the positive feedback they get from others. Although they hardly dare to ask for it, they definitely feel a need for it. An assignment that includes this in the instructions offers scope for it, or ‘legitimates’ the question.

Practice has shown that giving shape to a person’s *life story* is also a powerful intervention. Patients can create an expressive life line on which their entire development from birth to the present is represented in lines, shapes and colors showing the significance of certain periods (Haeyen, 2018a). Following on from this, they can zoom in on a particularly significant period. The images or ideas that arise can be presented in a personal manner. Besides a focus on profoundly negative periods, it is also important, certainly in the context of strengthening the healthy adult, to devote attention to significant positive experiences from the past. This allows patients to take a more balanced view of their life story. They can also reframe or relabel parts of their life story, as is done in schema therapy. Art therapists focus on formal elements such as color, dynamic, contour and so on, and associate balance, and specifically being out of balance, with the severity of the client’s problems and adaptability with the client’s strengths and resources (Pénzes et al., 2018).

Attention can also be focused on the time yet to come, on what people imagine or wish for the future. They can form a picture of the future to generate creativity and hope, as used in positive psychology. Or they can make a triptych showing past, present and future or a drawing of a metaphoric bridge between past and future (Darewych and Riedel Bowers, 2018). This can be approached as a strengths assessment in which patients portray an overview of their own sources of energy. It can even be structured in more detail by dividing it into different domains of life. Using the time categories past, present and future, earlier and present sources of energy find their place, as do hopes and aspirations for the future. This can be elaborated in images. Not all patients can express themselves well in words, and there are also patients whose words cannot reach their emotions. Working expressively offers these patients a different gateway.

The following quote shows how an intervention brings forward various aspects of the matters discussed above: practicing changing a behavioral pattern and the tendency to deprecate oneself, expressing one’s present experiential world and practicing self-compassion. For example, in art therapy a client can be asked to work with clay to create inside a bag and cannot see what (s)he is making, but works solely on the basis of touch.

“I like to work inside the bag because then no one can belittle the result! I can’t do a thing about it. Because I can’t see it, I can work more freely. What I made, what it turned into, is a sort of sea urchin skeleton. It represents how empty I often feel. I can be satisfied with the work, even though I see what is not yet “perfect” about it.” (woman, age 25)

Working solely on the basis of touch like this strenghtens the here and now experience, allows self expression without a judgemental stance. This is reinforcing and/or increasing the own sensoric expression of the inner experiential world.

### Art Mindfulness Exercises

Together with the assignments described above, art mindfulness exercises can be a good start for self-reflection. Rappaport (2009) stated that mindfulness practices could add a dimension of connecting the imaginal realm with the bodily felt experience to traditional art therapy, while the act of creating could offer a means of expressing the inner felt-sense when verbal expression proved inadequate or too difficult. Research to date has shown significant potential for mindfulness based art therapy, especially in areas such as stress reduction, social support, and emotional wellbeing (Hinchey, 2018).

Mindfulness and art-based therapies activate the same areas of the brain, so combining the two methods into one experience can come naturally for the human mind (Smalley and Winston, 2010). These art mindfulness exercises exercises make patients more aware of their own body, emotions and thoughts by calling directly on sensory experiences. By allowing abstract physical feelings to be expressed freely through art, as opposed to translating them into verbal communication, the client is able to stay as true to bodily sensations as possible, while still expressing them accurately (Rappaport, 2009). Working expressive, and the art materials themselves, stimulate insightful observation and tactile and physical perception. This calls for a complete, non-judgmental focus on a task or activity. The exercises are focused on perception in the here and now, not on the client’s past history. Examples of such exercises are targeted perception of the sound effect of tearing paper, or the flowing and mixing effect when applying diluted ink to damp paper. It can also involve attentive drawing of what is observed, or working on the basis of concentration and movement (Schweizer et al., 2009).

### Imagery Exercises

Schema-focused therapy for personality disorders works with experiential techniques, including imagery (Arntz and Bögels, 2000; Arntz, 2011, 2012; Reubsaet, 2018). Imagery is a technique used in cognitive therapy to clarify feelings and thoughts, to make links between recent events and experiences or perceptions from childhood, to lay bare dysfunctional basic schemas and to create newer, more functional schemas. Because imagery is primarily a non-verbal experience consisting of mental images, the newly created representations are felt more than thought. This is in keeping with personality disorders, which often have their origins in experiences from infancy and childhood, when the child had no-one to confide in and was even not yet able to use language, for example, to take in or respond to his mother’s reactions to him when he expressed his own emotions (Arntz and Bögels, 2000; Arntz, 2011, 2012). Using imagery, schemas can be felt, recognized and named. This brings insight into the personal history of a person’s schemas. At the same time new experiences are created, and new experiences allow the schema to change. What takes place during an imagery exercise is advised to be put into words and given meaning, so that the new information is processed at all levels. The focus in the imagery can also be rescripting the experience (Arntz, 2012) Hence, imagery exercises offer a context for working with concrete personal experiences and investigating and correcting the underlying schemas. Imagery is also used in art therapy. It provides a framework to work with concrete, personal experiences and to investigate the underlying schemas. Imagery is one of several art psychotherapeutic techniques that can open up deeper layers in the mind that can delve beyond verbal and concrete behavior. This is in part thanks to the fact that images belong to the inner domain, where people have the greatest degree of freedom and can be creative (e.g., Lusebrink, 1989; McNiff, 2001; Darewych and Riedel Bowers, 2018). After the imagery, the next step is giving an external shape to internal images using art materials. This allows patients to intensely feel these internal images, then anchor them, confirm them and reflect on them. Giving shape to what came up during the imagery allows patients to make their own choices and take some perspective, and calls on them to reflect further on the experiences. Here patients must be able to separate and distinguish past and present, and also to see links between present and past (Rubin, 2001; Schweizer et al., 2009; Haeyen, 2018a).

The findings in a study with a group of 48 patients who completed a questionnaire after an imagery exercise confirmed positive perceived effects and showed that the expressive aspect adds essential features. Making art work based on the imagery makes it possible to anchor, confirm and reflect on these images. An increase was seen in the happy child mode and the healthy adult, while the demanding parent, the angry child and the vulnerable child decreased (Haeyen, 2018a). To strengthen the Healthy Adult, the exercise must also focus on developing new behavior. The Healthy Adult is primarily seen as the mode in which, starting from problems and pathology, people make sensible choices to achieve change. During the imagery, patients may (for example) envision themselves acting as today’s adult in the difficult situation in which they were trapped as a child. The Healthy Adult takes the child’s need seriously and takes over management of the situation in a healthy adult manner. When these images are formed and expressed in a work of the client’s choice, it can have a strengthening and anchoring effect.

### Expressive Representation of the Various Modes

Art therapy is probably very effective in provoking experiences and feelings (mental states and promoting a healthy adult attitude to these feelings and experiences. This was the conclusion based on a pilot study conducted by Van den Broek et al. (2011) (*N* = 10), focused on the extent to which Schema Focused Therapy (SFT) and drama, music and arts therapies were able to provoke schema modes in forensic patients with a cluster B personality disorder. SFT was compared with treatment as usual (TAU) (i.e., cognitive behavioral therapy) and arts therapies were compared to psychotherapy. The comparison of psychotherapy to arts therapies showed a significant effect in relation to healthy modes (*d* = 0.80). Patients reported more healthy modes in art therapy groups than in psychotherapy (*T* = 7.00; *p* < 0.05). Art therapy that is part of TAU came forward as the most effective form of intervention.

**FIGURE 4:**
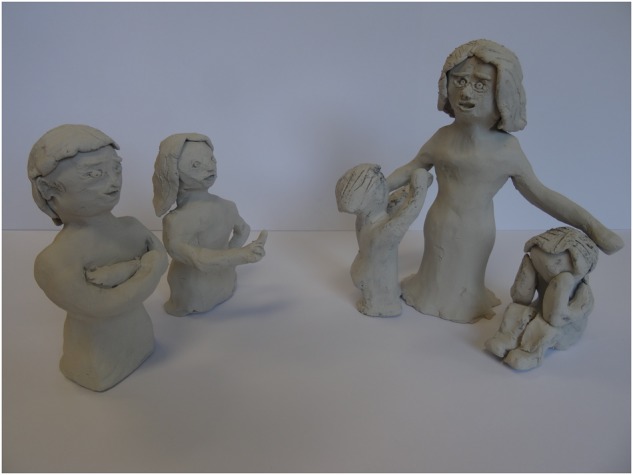
Configuration of modes.

Expressive representation of the various modes is often very elucidating – not only in terms of the nature of a mode, but also in the manner in which modes are related to each other. Patients can literally move around the mode images they have made until they stand in a correct relationship to each other (see Figure 4 and the accompanying quote). Working in this way is somewhat similar to role play or the multiple chair technique, but also has much overlap with Gestalt (Art) therapy (Moreno and Moreno, 1969; Rhyne, 1970, 2001). By leaving space between the modes, patients can work on an interpersonal dialogue. What do the various modes say to one another? What could you reply in mode x or y? These techniques are used so that patients will experience and express feelings and thoughts, clarify them, create links between recent events and experiences or perceptions from their childhood, and identify schemas. A new functional schema can take shape on the basis of what they are experimenting with and their ensuing experiences.

“So much is going on in my head. Each mode has its own voice and its own opinion. In order to get a better grasp of this, I took up a challenge to make a clay image of all the modes that play a role for me. The punitive parent, whose scolding finger is always ready to criticize and disapprove. The dominant, detached protector, ostensibly invulnerable and strong. The vulnerable child who puts out her arms for comfort and love but is not acknowledged. And the angry child, who is not allowed to show her presence and so, stores away the anger in herself. Each figure has its own story.

(…) the most important thing the healthy adult stands for is self-compassion. Amid all the strict and judgmental thoughts, that is what I’m looking for. A loving parent who allows the child modes to play a central role, allows the child to be angry or sad. A protector who protects the child modes against the punitive parent and the detached protector. The photograph still shows them in the line-up, but they are on the sidelines. I want to acknowledge that they are all there, okay, but I want to give them much less room. They will probably always be there, but now their role is too defining, they have too much control.” (woman, age 35)

Patients notice that, when they have a tangible picture of their modes and the role of the healthy adult, they manage to focus better on where they want to go. They state that if things are not going right and they tend to fall back in the role of the punitive parent, it is very helpful to think about the healthy adult who radiates compassion (Haeyen, 2018a). It helps to understand clearly which mode is actually talking and from there, to visualize what the healthy adult would say about this, how he or she would deal with it (Van Genderen and Arntz, 2010). The art medium helps to connect with these modes in an experientially way and by creating an art product the result is tangible and concrete (Gunther et al., 2009; Haeyen, 2018a).

### Interventions Focused on Developing Healthy Adult Patterns

The problems can often be discovered in how the patterns are given shape. For example, patterns can be recognized that are rigid, that set many high requirements, or that are impulsive and unstructured. In the art process, fixed patterns of cognitions, emotions and behaviors as well as the way a client interacts with the art media (e.g., controlled perfectionism, impulsive or affective behavior) are visible, according to many authors (e.g., Hinz, 2009; Schweizer et al., 2009). When these patterns are related to the problems and objectives people have, it offers excellent practice material. The results of a study by Haeyen et al. (2018) on client and art therapist perspectives (*N* = 11; 8 patients, three art therapists) showed that, with targeted use of visual art assignments and materials plus a personally chosen approach to art processes on the part of the client, linked to the Expressive Therapies Continuum (Hinz, 2009), patients experienced emotions, portrayed them and shared their experiences. In art therapy, the therapist can elicit a different manner of working. If there is a rigid pattern, the client’s spontaneity can be encouraged, taking small steps, by working with materials that are flowing or unstructured, such as clay, paints and liquid water colors (Hinz, 2009; Lusebrink, 1989). The therapist can also vary assignments and encourage spontaneity by setting a time limit, or working with scribbles or paint droplets. Patients who have problems with perfectionism are encouraged to lower the demands they make of themselves, to be satisfied with what they have achieved in a short period, and so on. This also becomes apparent when people see how others sometimes have a completely different way of dealing with things. An unstructured pattern without boundaries can be discouraged by setting requirements for planning skills, dealing with limitations in time and making more structured images (for example, by asking patients to make use of recognizable, structured forms rather than amorphous shapes) (Schweizer et al., 2009).

Part of a healthy adult pattern is making your own choices. A pattern of dependency and/or self-neglect is not foreign to many patients with a personality disorder. In working expressively, they must immediately start to make their own choices: they are in control of the design process. It is also appropriate to healthy adult behavior for people to try things out, practice letting go and reflect on sensory perceptions. These are bottom-up processes that refer to attention as driven mainly by the characteristics of the stimulus and its sensory context (e.g., contrast, symmetry, and order) (Yaniv, 2018). This means an open attitude to experiences in the present moment. This can be encouraged during the individual process of making something, but it can also be done by working expressively with others (for example, as a twosome or in the group). If patients tend to isolate themselves, then taking part and participating is very important. In the end, just sitting and watching does not work (Huckvale and Learmonth, 2009). While working together expressively, a sort of team spirit emerges.

The results of the earlier mentioned, recently conducted randomized controlled trial (RCT) show that art therapy is powerful in itself, not just as a supplement to a verbal therapy (Haeyen, 2018a; Haeyen et al., 2018a). The aim of this RCT (*N* = 74) was to evaluate the effects of a 10-weeks art therapy intervention for patients with a personality disorder of clusters B or C. In this study the Schema Mode Inventory was used, among other questionnaires. Compared to patients in the waiting list group, patients who received art therapy showed a decrease in personality disorder pathology, including maladaptive schema modes (less impulsivity, detachment, vulnerability and punitive behavior) and experiential avoidance (more acceptance of unpleasant inner experiences, such as thoughts, feelings and physical sensations). They also showed an increase in mental health functioning at a symptom-level and adaptive schema modes (pleasant feelings, spontaneity and self-regulation). The results of this RCT emphasized the potential of art therapy as an effective treatment option and showed its efficacy. Another quantitative study (*N* = 32) by Haeyen et al. (2018a) confirms that art therapy is an intervention that improves well-being and quality of life, in addition to the fact that it is a specific therapy to reduce specific symptoms. The results showed very large effect sizes on outcome measures for mental health.

### Interventions Focused on Limited Reparenting

Limited reparenting is also an important Schema-focused therapy technique (Arntz and Bögels, 2000). Schema therapy’s aim is to meet the needs by helping the patient find the experiences that were missed in early childhood that will serve as an antidote to the damaging experiences that led to maladaptive schemas and modes. Limited reparenting, paralleling healthy parenting, involves the establishment of a secure attachment through the therapist, within the bounds of a professional relationship, doing what he/she can to meet these needs. Art therapy adds extra ways and possibilities to achieve this goal. Limited reparenting in art therapy can be offered by interventions with the use of soft materials, by the use of supportive assignments that fit the needs of patients and by the therapist assuming a comforting role in the collaboration (e.g., Gunther et al., 2009; Haeyen et al., 2015. For example, the therapist can ask the patient to choose a soft textile to make a cuddle toy to keep with them and be warmly assisting in this process. Because the therapist always has materials, assignments and experiential techniques on hand, the relationship of patient to therapist is less direct; it also runs via the medium. The medium itself plays an important role: it evokes reactions and gives structure, feedback and consolation and so it works in the area of reparenting without any loss of the patient’s own responsibility. These reparenting strategies stimulate expression of feelings in the present moment, reflection on (contradictory) feelings and reappraisal, acceptance and integration of these feelings, corrective experiences, increased insight (Huckvale and Learmonth, 2009; Van Vreeswijk et al., 2012a).

## Conclusion

On the basis of our present knowledge, some available studies and practical experiences, the conclusion is that art therapy seems very promising in contributing to the development of Healthy Adult functioning. Art therapy addresses the six areas of healthy adult functions in patients diagnosed with personality disorders cluster B/C: (1) Personality integration and formation of self-image; (2) Healthy emotion regulation; (3) Coming in contact with one’s own feelings and needs as well as those of others; (4) The healthy internal dialogue; (5) Reality testing and assessment of situations, conflicts, relationships; and (6) Seeking enjoyment and need fulfillment in a mature manner. Art therapy can help to strengthen these healthy ego functions by specific art therapy interventions which are discussed on a concrete level with clear examples.

Art therapy focuses on healthy adult functioning from a positive psychology perspective and expands what is working in the patient’s life. Art therapy can be particularly well-suited to adapting to and aligning with the patient’s needs, starting from the positive basis of reliance on creativity and art materials. This revolves around questions such as: “What do you find agreeable? What could you make? How can you experiment and play around with possibilities in which the positive challenge forms the basis for further development? What are your possibilities, and what is impossible for you, and with this in mind, how can you shape something that is meaningful for you?” In art therapy, complaint-oriented and strength-oriented work often go hand in hand: working with a focus on strength by taking as the basis an affinity with the activity, the painting, drawing or spatial design, and working with a focus on complaints by practicing things that are difficult, resolving roadblocks by getting moving both literally and figuratively. Working with objectives and expressing your inner world, which is often fraught with conflicts, can focus on strengths and complaints at the same time. Art therapy has the capacity to add to the patient’s strength, to arouse experiences of flow and positive emotions and to express positive emotions and meaning (Wilkinson and Chilton, 2013).

However, more research is needed to verify the effects and working mechanisms of art therapy. The specific art therapy interventions should be quantitatively researched. Future research could investigate the effectiveness of these interventions and the validity of the assumptions that are made. The mentioned specific art therapy interventions can also help to choose a focus for future studies. Experiential techniques have recently received more attention from verbal therapies. They are increasingly being included in a range of cognitive behavioral therapies (such as schema-focused therapy (SFT) or acceptance and commitment therapy (ACT), and are mainly used to supplement a central basis of verbal interventions. Arntz (2011, 2012), for example, states that these experiential techniques (used as a part of Schema Focused Therapy) hold a promise for therapeutic use, although he emphasizes the need for systematic empirical evaluation of these techniques and to unravel underlying mechanisms of change (Haeyen, 2018a).

The strength of art therapy may well be the activities, experiences and effects that appeal to patients on an activity or experiential level, sometimes typified as “in the place of, alongside and beyond words.” The therapist sees, and within the therapy situation, the patient sees and perceives possibilities and relationships that are often difficult to express in everyday language. The non-verbal experiential background that people build up in the course of their life has a highly determining effect on their behavioral repertoire, their manner of affectively experiencing, cognitive framing and interpreting events in relation to themselves and their surroundings. Using targeted interventions, the art therapist tries to nourish and stimulate these often deeply rooted experiences, or to encourage patients to seek alternatives. Because their starting point is the activity or experiential level, these experiences are sometimes easier to reach, and developing from there into a Healthy Adult mode is possible in a manner that is more felt than thought.

If patients have no motivation or no clearly defined request for help, or if they show resistance to the experience, the art therapist can make use of motivational techniques and the indirect aspects offered by the medium. The art therapist can make use of a wide range of media and techniques to align and coordinate, but also to encourage directive schemas or modes, to make them part of the patient’s experience and where possible, to add a bit of extra color or bring about a change. Art therapy can use more broadly based interventions, and can look further than the complaint or problem. Its strength lies in making use of the possibilities patients already have, of their subconscious competence and their capacity to play, to move and change, and to be creative. From this viewpoint, creativity can also be regarded as the ability of the Healthy Adult to be flexible and to find different solutions to a problem. This process often takes place without the use of language: it is more felt than thought. This broader appeal of art therapy to strong, healthy and adult aspects of the person has a confirming effect that fits well to a therapeutic principle such as empowerment and the patient’s well-being, which is at the heart of positive psychology.

“The creative adult is the child who has survived.” (Le Guin, 2014)

## Ethics Statement

An ethics approval was not required as per applicable institutional and national guidelines and regulations. All participants provided written informed consent both for the purposes of research participation as well as for the publication of their art products in the images of the manuscript in accordance with the Declaration of Helsinki.

## Author Contributions

The author confirms being the sole contributor of this work and has approved it for publication.

## Conflict of Interest Statement

The authors declare that the research was conducted in the absence of any commercial or financial relationships that could be construed as a potential conflict of interest.
